# A novel acute HIV infection staging system based on 4^th^ generation immunoassay

**DOI:** 10.1186/1742-4690-10-56

**Published:** 2013-05-29

**Authors:** Jintanat Ananworanich, James LK Fletcher, Suteeraporn Pinyakorn, Frits van Griensven, Claire Vandergeeten, Alexandra Schuetz, Tippawan Pankam, Rapee Trichavaroj, Siriwat Akapirat, Nitiya Chomchey, Praphan Phanuphak, Nicolas Chomont, Nelson L Michael, Jerome H Kim, Mark de Souza

**Affiliations:** 1 SEARCH, 104 Tower 2, 2nd Floor, Rajdamri Road, Pathumwan, Bangkok, 10330, Thailand; 2 The Thai Red Cross AIDS Research Centre, 104 Rajdamri Road, Pathumwan, Bangkok, 10330, Thailand; 3 HIV-NAT, 104 Rajdamri Road, Pathumwan, Bangkok, 10330, Thailand; 4 Department of Medicine, Faculty of Medicine, Chulalongkorn University, 1873 Rama IV, Pathumwan, Bangkok, 10330, Thailand; 5 Vaccine and Gene Therapy Institute Florida, 9801 SW Discovery way, Port St. Lucie, FL, 34987, USA; 6 Department of Retrovirology, Armed Forces Research Institute of Medical Sciences – US Component, 315/6 Rajvithi Rd., Bangkok, 10400, Thailand; 7 US Military HIV Research Program, 503 Robert Grant Ave, Silver Spring, MD, 20910, USA

**Keywords:** Acute HIV infection, Primary HIV infection, Fiebig stage, 4thG stage, Enzyme immunoassay, 4^th^ generation EIA, 2^nd^ generation EIA, Nucleic acid testing, Reservoir, Functional cure

## Abstract

**Background:**

Fourth generation (4thG) immunoassay (IA) is becoming the standard HIV screening method but was not available when the Fiebig acute HIV infection (AHI) staging system was proposed. Here we evaluated AHI staging based on a 4thG IA (4thG staging).

**Findings:**

Screening for AHI was performed in real-time by pooled nucleic acid testing (NAT, n=48,828 samples) and sequential enzyme immunoassay (EIA, n=3,939 samples) identifying 63 subjects with non-reactive 2^nd^ generation EIA (Fiebig stages I (n=25), II (n=7), III (n=29), IV (n=2)). The majority of samples tested (n=53) were subtype CRF_01AE (77%). NAT+ subjects were re-staged into three 4thG stages: stage 1 (n=20; 4^th^ gen EIA-, 3^rd^ gen EIA-), stage 2 (n=12; 4^th^ gen EIA+, 3^rd^ gen EIA-), stage 3 (n=31; 4^th^ gen EIA+, 3^rd^ gen EIA+, Western blot-/indeterminate). 4thG staging distinguishes groups of AHI subjects by time since presumed HIV exposure, pattern of CD8+ T, B and natural killer cell absolute numbers, and HIV RNA and DNA levels. This staging system further stratified Fiebig I subjects: 18 subjects in 4thG stage 1 had lower HIV RNA and DNA levels than 7 subjects in 4thG stage 2.

**Conclusions:**

Using 4^th^ generation IA as part of AHI staging distinguishes groups of patients by time since exposure to HIV, lymphocyte numbers and HIV viral burden. It identifies two groups of Fiebig stage I subjects who display different levels of HIV RNA and DNA, which may have implication for HIV cure. 4^th^ generation IA should be incorporated into AHI staging systems.

## Findings

Acute HIV infection (AHI) refers to the initial period of rapid and widespread destruction of immune cells and uncontrolled viremia after HIV acquisition. This period usually lasts about 4 weeks, during which HIV infectiousness is the highest [1]. Thus identification of AHI has direct implications for HIV treatment and prevention [2]. Enhanced recovery of CD4+ T cells, preservation of immunity and reduction of HIV reservoir size have been shown when antiretroviral treatment (ART) is initiated during AHI [3-5], which may afford patients higher response to future HIV cure strategies [6]. The contribution of AHI to new HIV transmission is substantial, and elimination of HIV spread requires ART in AHI in addition to chronic HIV infection [1,7]. AHI is characterized by positive HIV nucleic acid testing (NAT) and a non-reactive or indeterminate Western blot (WB) [8]. The 2^nd^ generation (2ndG) enzyme immunoassay (EIA) detects IgG to HIV, and was, until recently, used as standard screening in HIV diagnostic algorithms. However, the lag time between infection and reactivity (window period) is 25–35 days [9] The 4^th^ generation (4thG) antigen-antibody combination EIA which detects p24 antigen, HIV IgM and IgG antibodies and shortens the window period to 15–20 days is now replacing the 2ndG EIA.

Staging of AHI provides a temporal framework for the earliest events in HIV infection, permitting meaningful comparison of observations regarding pathogenesis and reservoirs, stratification of cohorts, and extrapolation of outcomes [10]. Persons with AHI who initiate ART before peak viremia when IgM antibody to HIV is still non-reactive appear to exhibit favorable immunologic and virologic outcomes [11-13]. The current AHI staging system was published by Fiebig and colleagues in 2003 prior to 4thG EIA availability [14] and incorporates 2ndG EIA as part of the staging algorithm. However, commercial 2ndG EIA test kit production and use in the US have been drastically reduced and is no longer available in many parts of the world including Thailand. Here we propose a staging system that uses 4thG EIA (4thG staging) by examining data from the RV254/SEARCH 010 study, in which samples from HIV testing clients in Bangkok, Thailand, are screened to identify AHI. Fiebig I to IV AHI subjects are offered enrollment and ART (clinicaltrials.gov identification numbers NCT00796146 and NCT00796263). The study was approved by the Chulalongkorn University Institutional Review Board (IRB) in Thailand, and at 3 IRBs in the US including the Walter Reed Army Institute of Research, the University of California at San Francisco, and Yale University

Between 04/2009 to 04/2012, 52,767 samples were first screened with 4thG EIA (AxSYM, Abbott Laboratories, Wiesbaden, Germany or Roche HIV Combi Assay; Roche Diagnostics, London, UK). Non-reactive samples were screened by pooled NAT (n=48,828) using either Roche Amplicor v 1.5 assay with a detection limit of 50 copies/ml (Roche Diagnostics, Branchburg, NJ, USA) or Aptima HIV-1 RNA assay with a detection limit of 30 copies/ml (Gen-Probe Inc., San Diego, CA, USA). For reactive 4thG EIA (n=3,939) samples, the 2ndG EIA was performed. AHI was confirmed by a non-reactive 2ndG EIA and a second positive HIV RNA. An IgM-sensitive (3^rd^ generation, 3rdG) EIA (Genscreen HIV 1/2, Bio-Rad, Marnes la Coquette, France), HIV-1 p24 antigen assay (ABL Inc., Kensington, MD) without immune-complex dissociation and WB were done for Fiebig staging according to published criteria [14]. 4thG staging used 4thG and 3rdG EIA, and NAT results to identify 3 AHI stages: stage 1 (4thG EIA-, 3rdG EIA-), stage 2 (4thG EIA+, 3rdG EIA-) and stage 3 (4thG EIA+, 3rdG EIA+, WB-/indeterminate). We estimated the time from history of HIV exposure within the last 30 days to the baseline visit for each subject. For subjects who had multiple dates for possible HIV exposure, the average time from exposure was used. 4thG EIA testing was performed on fresh samples, and all other tests were performed on fresh plasma or sera or stored at ≤ -80°C with a single freeze-thaw cycle. Immunophenotyping of peripheral blood mononuclear cells (PBMCs) for CD4+ and CD8+ T cells, CD19+ B cells, CD16+CD56+ natural killer cells, and determination of total and integrated HIV DNA were performed according to published methods [4,15]. All tests with the exception of 4thG EIA were performed in a College of American Pathologists-accredited clinical laboratory (AFRIMS Retrovirology Clinical Laboratory, Bangkok). The 4thG EIA was performed at a nationally recognized laboratory that is part of the Thai Red Cross Blood Banking laboratory network. HIV subtyping was determined by the multi-region hybridization assay [16]. Data were summarized by number and percentage for categorical variables, median and inter-quartile range (IQR) for continuous variables. Pairwise comparisons between AHI staging were done by Mann–Whitney U test. Statistical analyses were performed using Prism version 5.01 software (Graphpad, software inc.) and STATA/IC version 11.2 for windows (Statacorp LP, TX, USA).

Of 52,767 samples screened, 89 AHI subjects were identified and 75 enrolled in the study. Fourteen did not enroll because 2 did not want to enroll, 5 could not be contacted, 4 were not Thai and 3 no longer had AHI. Twelve were further excluded from the analysis: 6 had reactive 2ndG EIA at enrollment and 6 gave discordant results between the 4thG and the 3rdG EIA for which technical error could not be ruled out. Of 63 subjects included in this analysis, 57 were men who have sex with men. The median age was 29 years. HIV-1 subtyping was performed on 53 samples; 41 (77%) were CRF_01AE, 4 (8%) were CRF_01AE/B, 1 (2%) was B and 7 (13%) were non-typable. Characteristics by Fiebig and 4thG staging at time of AHI diagnosis and at 24 weeks of ART are shown in Table 1. Similar to Fiebig staging, differences were observed between 4thG stage 1 and later stages in time since HIV exposure, p24 antigen and HIV RNA and DNA levels. The time since history of HIV exposure in 4thG stage 1 was shorter than 4thG stage 2 by 5 days and 4thG stage 3 by 6 days. Compared to Fiebig I stage, 4thG stage 1 had slightly shorter time since HIV exposure and lower HIV RNA levels. Eighteen in Fiebig 1 (72%) were in 4thG stage 1 and 7 (28%) in 4thG stage 2. These two groups in Fiebig I were different in that those in 4thG stage 1 had lower HIV RNA and HIV DNA, and tended to have a shorter time since exposure (Table 2).

**Table 1:** Characteristics of subjects by Fiebig and 4th generation enzyme immunoassay staging system at the time of acute HIV infection diagnosis and week 24 after initiating antiretroviral therapy

	**Fiebig stages**				**4thG stages**		
	**Stage I**	**Stage II**	**Stage III**	**Stage IV**	**Stage 1**	**Stage 2**	**Stage 3**
	**NAT+/p24-/3**^**rd **^**G-(n=25)**	**NAT+/P24+/3**^**rd **^**G-(n=7)**	**NAT+/p24±/3**^**rd **^**G+/WB-(n=29)**	**NAT+/p24±/3**^**rd **^**G+/WB IND (n=2)**	**NAT+/4**^**th **^**G-/3**^**rd **^**G-(n=20)**	**NAT+/4**^**th **^**G+/3**^**rd **^**G-(n=12)**	**NAT+/4**^**th **^**G+/3**^**rd **^**G+/WB- or IND (n=31)**
**At time of acute HIV infection diagnosis (n=63)**							
Median (IQR) days from history of HIV exposure	14 (9–18)	15 (11–17)	18* (13–22)	26 (NA)	12 (9–15)	17* (15–21)	18* (13–22)
Range (Min-Max)	(4–40)	(9–18)	(9–33)	(20–32)	(4–40)	(10–34)	(9–33)
Median (IQR) HIV RNA, log_10_copies/mL	5.1 (4.1-5.4)	5.8 * (5.1-6.5)	5.9 ** (5.6-6.9)	5.6 (NA)	4.8 (3.7-5.4)	5.8** (5.4-6.2)	5.8** (5.6-6.9)
Range (Min-Max)	(2.8-6.1)	(5.1-7.6)	(4.7-7.7)	(5.5-5.8)	(2.8-5.7)	(5.1-7.6)	(4.7-7.7)
Median(IQR) p24 pg/mL	3.9 (0–20.1)	190** (124–794)	262** (46.1-937.2)	106 (NA)	0.3 (0–27.4)	54.3* (14.7-203.5)	227.3** (42.6-937.2)
Range (Min-Max)	(0–65)	(74.3-862)	(15.7-6973.9)	(16.9-195)	(0–133.9)	(3.7-862)	(15.7-6973.9)
Median(IQR) CD4 cells/mm^3^	413 (311–565)	289 (218–426)	381 (298–428)	371 (NA)	451 (316–592)	316 (265–420)	381 (295–463)
Range (Min-Max)	(214–1127)	(179–569)	(132–621)	(279–463)	(218–1127)	(179–698)	(132–621)
At week 24 after antiretroviral therapy (n=61, excluded 2 subjects who did not start treatment)*							
Median (IQR) days from diagnosis to ART initiation	2 (2–3)	2 (1–2)	2 (1–3)	2 (NA)	2 (2–4)	2 (1–2)	2 (1–3)
Range (Min-Max)	(0–5)	(0–2)	(0–5)	(1–3)	(1–5)	(0–4)	(0–5)
Median (IQR) HIV RNA	1.7	1.7	1.7	1.7	1.7	1.7	1.7
log_10_copies/mL	(1.7-1.7)	(1.7-1.7)	(1.7-1.7)	(NA)	(1.7-1.7)	(1.7-1.7)	(1.7-1.7)
Range (Min-Max)	(1.7-1.7)	(1.7-2.0)	(1.7-2.2)	(1.7-1.7)	(1.7-1.7)	(1.7-2.0)	(1.7-2.2)
Median change (IQR) HIV RNA log_10_copies/mL	-3.4 (-3.7 to -2.4)	-3.8* (-4.7 to -37)	-4.2 ** (-5.2 to -3.9)	-3.9 (-4.1 to -3.8)	-3.0 (-3.7 to -1.9)	-4.0* (-4.4 to -3.7)	-4.1** (-5.2 to -3.8)
Range (Min-Max)	(-4.4 to -1.1)	(-4.8 to -3.4)	(-6.0 to -3.0)	(-4.1 to -3.8)	(-4.0 to -1.1)	(-4.8 to -3.4)	(-6.0 to -3.0)
Median (IQR) CD4 cells/mm^3^	600 (540–904)	766 (523–772)	579 (486–730)	821 (NA)	597 (483–794)	904* (556–1056)	579 (470–765)
Range (Min-Max)	(312–1084)	(354–1145)	(301–1229)	(462–1180)	(312–979)	(503–1145)	(301–1229)
Median change (IQR) CD4 cells/mm^3^	256 (50–358)	344 (203–477)	227 (180–320)	450 (NA)	136 (-25 to 280)	477** (291–719)	227* (180–347)
Range (Min-Max)	(-237-766)	(136–719)	(-135-804)	(183–717)	(-237-383)	(203–766)	(-135-804)
Nonreactive 2^nd^ generation EIA, N	10	0	4	1	9	1	5
Nonreactive/IND WB, N	11	0	4	1	11	0	5

P value compared to stage 1 within each staging system: *p < 0.05, **p < 0.001 All patients had non-reactive 2^nd^ generation enzyme immunoassay, negative/indeterminate Western Blot and positive nucleic acid testing.

Abbreviations used: *NAT* nucleic acid testing; 3^rd^*G* 3^rd^ generation enzyme immunoassay; 4^th^*G* 4^th^ generation enzyme immunoassay; *EIA* enzyme immunoassay; *WB* Western blot; *IND* indeterminate; *IQR* inter-quartile range; *NA* not applicable.

*Two patients did not start ART; they were both in Fiebig II stage while one was in 4thG stage1 and the other in 4thG stage2.

**Table 2:** 4thG staging distinguished two groups of Fiebig stage I acute HIV infection subjects

**Characteristics of Fiebig I subjects (n=25)**	**4thG stage 1**	**4thG stage 2**	**P value**
	**(n=18)**	**(n=7)**	
HIV RNA, log_10_copies/ml	4.6	5.7	0.005
HIV DNA, copies/10^6^ PBMC	7	312	0.002
Duration since history of HIV exposure, days	12.5	17	0.06

Abbreviation: *PBMC* peripheral blood mononuclear cells.

**Figure 1:**
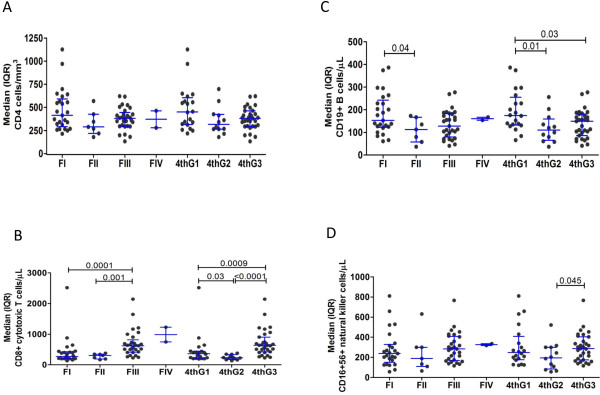
**Frequency of cell subsets in the peripheral blood at time of acute HIV infection using Fiebig and 4**^**th **^**generation enzyme immunoassay staging systems for CD4+ T cells (A), CD8+ T cells (B), CD19+ B cells (C) and CD16+56+ natural killer cells (D).** FI to IV are Fiebig acute HIV infection stages I to IV. 4thG 1 to 3 are 4thG acute HIV infection stages 1 to 3. The whiskers indicate median (inter-quartile range).

CD4 values were not significantly different between stages for both systems (Figure 1A). Both staging systems were able to discern differences in the CD8+ T cells (Figure 1B) and B cells (Figure 1C) between stages, although the sample size is relatively small particularly for Fiebig II. In addition, natural killer cells (Figure 1D) were different between 4thG stages 2 and 3.

All groups started ART about 2 days after enrollment (Table 1). At 24 weeks of ART, the HIV RNA suppression was similar across groups. 4thG stage 1 had smaller changes from baseline of HIV RNA, HIV DNA, and CD4 than the later stages, possibly as a result of difference in baseline values. About half of persons in Fiebig I (10/25) and 4thG stage 1 (9/20) had non-reactivity to both 2ndG EIA and WB at week 24 of ART.

**Figure 2:**
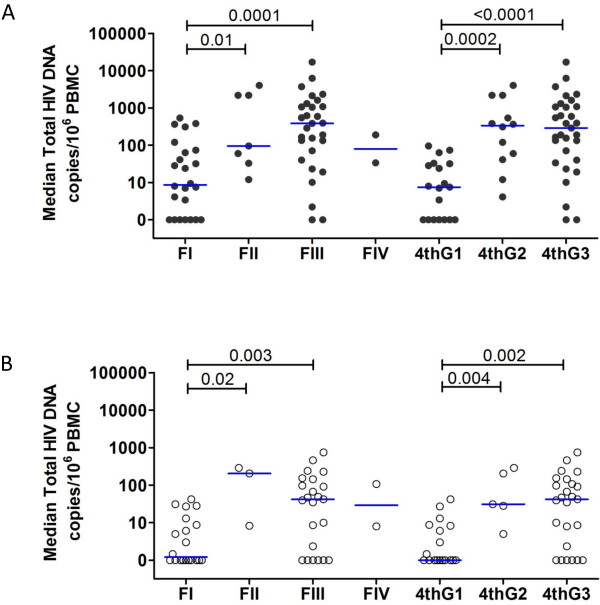
**Reservoir size in the peripheral blood at time of acute HIV infection (A) and at 24 weeks of antiretroviral therapy (B) using Fiebig and 4th generation enzyme immunoassay staging systems.** FI to IV are Fiebig acute HIV infection stages I to IV. 4thG 1 to 3 are 4thG acute HIV infection stages 1 to 3. The whiskers indicate median (inter-quartile range).

The total HIV DNA values in PBMCs at time of AHI (Figure 2A) and at 24 weeks of ART (Figure 2B) were significantly lower in the earliest AHI stages with both staging systems. The undectectable total HIV DNA despite the presence HIV viremia at time of AHI diagnosis (Figure 2A) suggest that in the very early stages of AHI, HIV production may primarily occur at secondary lymphoid tissues such as in the lymph nodes as previously reported in macaques and humans [17-19].

These data suggest that 4thG staging can distinguish groups of persons in AHI by time since HIV infection, dynamics of lymphocyte subsets and HIV viral burden. The dynamics of CD4+ T, CD8+ T, B and NK cells in the three 4thG stages correspond with findings from RV217, a study that used biweekly small volume NAT in high-risk populations, and serially documented lymphocyte dynamics from before HIV infection through the AHI in Thais and Africans [20]. Such cellular dynamics may have importance in viral control but the relative contribution of each cell subset is yet to be determined in humans [21]. The current study showed 100-fold higher median HIV RNA for subjects in Fiebig I compared to the original Fiebig study, which may be due to differences in the predominant HIV subtype (CRF01_AE vs. B) [14].

We have previously shown that gut T cell depletion and HIV DNA reservoir size increased as Fiebig stage progressed [4]. Importantly, persons in Fiebig I displayed gut CD4+CCR5+ T cell preservation at levels seen in uninfected subjects. Persons in Fiebig I have extremely low total HIV DNA levels and almost all have undetectable integrated HIV DNA in PBMCs [22]. Using 4thG staging, it was possible to further characterize persons in Fiebig I into those with nonreactive and reactive 4thG EIA (stages 1 and 2, respectively). These subjects differ by viral and pro-viral burden. This may have implications for HIV cure strategies as levels of HIV DNA predicts ability to control viremia when ART initiated during AHI is interrupted (functional cure) [23,24]. The responses after ART are similar between the two staging systems. 4thG stage 1 group had the highest frequency of non-reactivity to HIV IgG detection assays. Together with low HIV DNA levels, this suggests that these patients have a distinctly low HIV reservoir size and viral burden.

The 4thG staging has some limitations. Different NAT and IA methods/kits may not have the same detection thresholds, which could reduce cross-study comparability. Although 4thG staging eliminates the need for 2ndG EIA, it does require WB (4thG stage 3), which may reduce its applicability in resource-limited settings. However, WB is not required to identify the earlier stages of AHI (4thG stages 1 and 2), and these stages may be most relevant to HIV cure. With low HIV reservoir size, these people are among the best candidates for future HIV cure strategies. It will be important to determine the predictive abilities of 4thG stages 1 and 2 on HIV reservoir characteristics and HIV-specific immunity that may impact response to interventions and, ultimately, HIV functional cure, in future studies.

## Abbreviations

AHI: Acute HIV infection; EIA: Enzyme immunoassay; IA: Immunoassay; WB: Western blot; 4thG: 4^th^ generation; 3rdG: 3^rd^ generation; 2ndG: 2^nd^ generation; NAT: Nucleic acid testing; ART: Antiretroviral therapy; PBMC: Peripheral blood mononuclear cells

## Competing interests

The authors declare that they have no competing interests.

## Authors’ contributions

JA, JLKF, SP, AS, JHK, MdeS conceived and designed the study, and drafted the manuscript. FVG, NC, PP and NLM participated in the study design, coordination and data interpretation. TP, RT, SA participated in the acute HIV infection testing and its interpretation. AS, CV and NC participated in the testing and interpretation of immunophenotyping and HIV DNA quantification. SP performed the statistical analysis. All authors read, provided input in the manuscript and approved the final manuscript.

## Disclaimer

This work has been presented at the 19th Conference on Retroviruses and Opportunistic. March 3–6, 2013, Atlanta (Poster 634).

The RV254/SEARCH 010 Study Group includes from *SEARCH/TRCARC/HIV-NAT*: Nipat Teeratakulpisarn, Nittaya Phanuphak, Eugene Kroon, Duanghathai Sutthichom, Somprartthana Rattanamanee, Peeriya Mangu, Michittra Boonchan, Supanit Pattanachaiwit; from *AFRIMS*: Viseth Ngauy, Vatcharain Assawadarachai; from *the US Military HIV Research Program*: Sodsai Tovanabutra, Merlin Robb; from *the Vaccine and Gene Therapy Institute-Florida*: Rafick Sekaly.
